# Influence of dendritic polyglycerol sulfates on knee osteoarthritis: an experimental study in the rat osteoarthritis model

**DOI:** 10.1186/s12891-015-0844-3

**Published:** 2015-12-15

**Authors:** Tobias Schneider, Pia Welker, Kai Licha, Rainer Haag, Gundula Schulze-Tanzil

**Affiliations:** Department of Orthopaedic, Trauma and Reconstructive Surgery, Charité-Universitätsmedizin Berlin, Campus Benjamin Franklin, Berlin, Germany; IC Discovery GmbH, Robert Koch Platz 4, Berlin, 10115 Germany; Institute for Chemistry and Biochemistry, Freie Universität Berlin, Takustrasse 3, Berlin, 14195 Germany; Institute of Anatomy, Paracelsus Medical University, Salzburg and Nuremberg, Germany, Prof. Ernst Nathan Str. 1, Nuremberg, 90419 Germany

**Keywords:** Dendritic polyglycerol sulfates, Osteoarthritis, Rat model, Chondrocyte, Nanoparticular compounds

## Abstract

**Background:**

Anti-inflammatory nanoparticular compounds could represent a strategy to diminish osteoarthritis (OA) progression.

The present study was undertaken to prove the uptake of nanoparticular dendritic polyglycerol sulfates (dPGS) by rat-derived articular chondrocytes and to answer the question of whether dPGS could modulate knee joint cartilage degradation in a rat OA model and whether complications could arise.

**Methods:**

dPGS uptake and cytotoxicity was assessed in cultured primary rat-derived articular chondrocytes. Subsequently, OA was induced in the right knee joints of 12 male Wistar rats by medial collateral ligament and meniscus transection. Unoperated left knees remained as controls. Six weeks post surgery six rats were either treated daily (14 days) with 30 mg/kg dPGS (s.c.) or a similar volume of physiological saline. Animals were analyzed clinically for gait alterations. Explanted knee joints were studied histologically using OA scores according to Mankin (1971), Glasson et al., (2010) and the synovitis score according to Krenn et al., (2006). Liver, spleen and kidneys were analyzed for degenerative changes due to dPGS accumulation.

**Results:**

dPGS was taken up after 2 hours by the chondrocytes. Whereas no significant clinical signs of OA could be detected, at the histological level, all operated rat knee joints revealed features of OA in the medial compartment. The values produced by both OA score systems were lower in rats treated with dPGS compared with saline-treated animals. Synovitis score did not significantly differ between the groups. The analyzed organs revealed no degenerative changes.

**Conclusions:**

dPGS presented overall cyto- and biocompatibility, no accumulation in metabolizing organs and chondroprotective properties in the osteoarthritic knee joint.

**Electronic supplementary material:**

The online version of this article (doi:10.1186/s12891-015-0844-3) contains supplementary material, which is available to authorized users.

## Background

OA development and progression is associated with inflammatory events affecting chondrocytes and synovial fibroblasts. Osteoarthritis (OA) is highly prevalent in Western society today, with more than two third of people over 65 years old with osteoarthritic alterations in their joints [1]. Besides age, other risk factors associated with OA such as obesity, traumatic cartilage injury, genetics, and muscle weakness are well known [2]. Pro-inflammatory cytokines such as tumor necrosis factor alpha (TNFα) or interleukin 1 (IL-1) are elevated in synovial fluids of OA patients [3]. The increased production of catabolic enzymes, mediated by pro-inflammatory cytokines, and impaired extracellular matrix de novo synthesis facilitates destruction of cartilage extracellular matrix, over time. Therefore, experimental strategies should be explored, which might be able to shift that imbalance back in a more favorable state. Nanoparticular compounds have not only been studied as potential drug delivery systems [4] but also have been proven to be therapeutically effective [5, 6]. Dendritic polyglycerols can be coupled with sulfate ions by substituting prior attached hydroxyl groups leading to dendritic polyglycerol sulfates (dPGS) which possess anti-inflammatory abilities, for example they reduce the migration of white blood cells.

In a previous *in vitro* study dPGS showed no cytotoxicity on chondrocytes and penetrated to some degree (50 μm) through the cartilage extracellular matrix [7]. It could upregulate anti-inflammatory IL-10 and modulate pro-inflammatory TNFα expression [7].

In the present work rat-derived articular chondrocytes were characterized and treated with dPGS *in vitro* to study its uptake. Further, OA was surgically induced in the right knees of adult Wistar rats before treated with dPGS to answer the following questions. 1. Do cultured rat-derived articular chondrocytes take dPGS up? 2. Are dPGS biocompatible? 3. Does dPGS modulate knee joint inflammation and cartilage degradation in a rat OA model? 4. Does dPGS accumulate during a treatment for two weeks and does it lead to complications *in vivo*? For answering the second question histological OA scoring served as an endpoint. To study putative side effects of dPGS accumulation main metabolic, excretory and immune organs were analyzed histopathologically.

## Methods

### Isolation and culturing of rat chondrocytes

Three adult male Wistar rats (Charles River Laboratories, Germany) were euthanized and cartilage slices were shaved from the articular surface of both knee joints. All the procedures with the rats were approved by the local animal ethics committee of Berlin, Germany (registration number G0159/11 and T0224/08). Afterwards, pooled cartilage slices were incubated in 1.5 mL pronase (2 % [Serva Electrophoresis GmbH, Germany]) solution for 20 minutes at 37 °C. The remaining pure cartilage was rinsed 2× with phosphate buffered saline (PBS). Subsequently 1.5 mL of collagenase solution was added and the sample was incubated again at 37 °C until the extracellular matrix was completely digested. Thereafter, cells were counted and seeded for future experiments in T-25 cell culture flasks (Sarstedt AG, Germany).

### Immunofluorescence labelling of cultured primary articular chondrocytes

The chondrocytes were cultured on cover slips for 48 hours before fixed using 4 % paraformaldehyde solution (PFA [Santa Cruz Biotechnology, Inc., USA]). The cells were treated with blocking solution consisting of 5 % donkey serum in Tris-buffered Saline (TBS) and 0.1 % Triton (Sigma Aldrich, Germany). Subsequently, the chondrocytes were incubated with the polyclonal rabbit-anti-human type II collagen, type I collagen antibodies (both 1:60, Acris Antibodies, Germany), Sox9 antibody (Chemicon, USA) or monoclonal mouse-anti-human vinculin antibody (Sigma Aldrich) overnight at 4 °C. Thereafter the cover slips seeded with chondrocytes were washed with TBS and incubated with the secondary antibody (donkey-anti-rabbit or donkey-anti-mouse IgG) coupled with Alexa-488 (1:200, Life Technologies, USA) or Cy3 (Invitrogen, USA) and 4’, 6-diamidino-2-phenylindole dihydrochloride (1:200, DAPI [Roche Applied Science, Germany]) for 1 hour at room temperature (RT).

### Uptake and cellular localization of dPGS nanoparticular compounds

The articular chondrocytes of the three rats (passage 3) were cultured on poly-L-lysine coated cover slips (VWR International, Germany) for 24 hours. Subsequently, cells were stimulated for 2 hours to 7 days with dPGS at a concentration of 10^−6^ mol/L which was linked with ICC (Indocarbocyanine, mivenion GmbH, Germany). At each point in time the cells were fixed using 4 % PFA and then stained with DAPI (Sigma Aldrich) to visualize the cell-nuclei and phalloidin-FITC (Sigma Aldrich) to depict the actin cytoskeleton. Images were taken using the fluorescence microscope Axioskop 40 (Zeiss AG, Germany) equipped with an Olympus XC30 camera (Olympus Soft Imaging solutions GmbH, Germany).

### FACS (fluorescence activated cell sorting) analysis of uptake and release of dPGS in rat chondrocytes

The pool of rat articular chondrocytes was seeded at a density of 20.000 cells/cm^2^ in T-25 cell culture flasks (passage 4). After 24 hours the cells were either treated with dPGS-ICC or as a control with glycerol-ICC (both groups: 10^−6^ mol/L, mivenion GmbH, Germany, for 2 hours, 72 hours and 7 days). One flask remained untreated as control. Then, trypsinized cells were fixed with 4 % PFA and resuspended in FACS buffer. 10.000 events per sample have been counted using a FACSCalibur machine (Becton, Dickinson and Company, USA) and data was analyzed using the FlowJo Software (version 7.6, Tree Star, Inc., USA).

### OA induction in the rat model

**Fig. 1:**
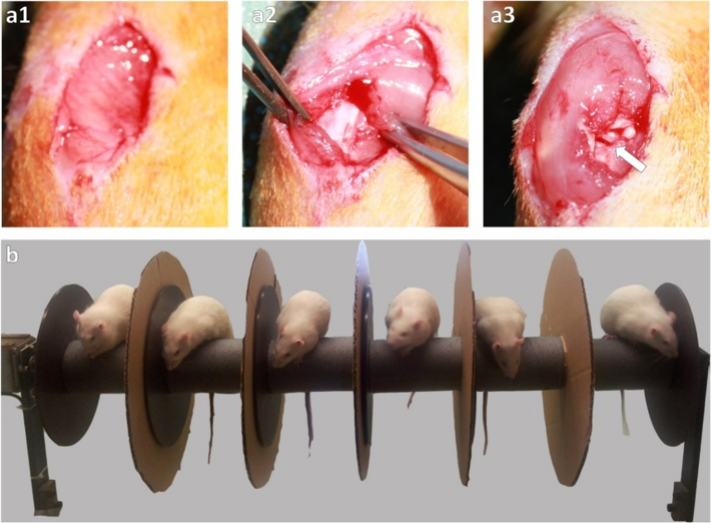
**a**-**b** OA induction in the Wistar rat model and forced mobilization apparatus. Twelve male Wistar rats underwent surgical destabilization of the right knee joint in response to dissection of the *ligamentum collaterale mediale* and *meniscus medialis* leading to OA. **a1**: skin incision, **a2**: *ligamentum collaterale mediale*, **a3**: joint space (arrow). To ensure OA development the rats started one week after surgery a daily training using a forced rotor disc mobilization device (**b**)

To assess the effects of dPGS on injured cartilage and joint tissue *in vivo* OA was surgically induced in the knee joints of 12 adult male Wistar rats (Charles River Laboratories, Germany). The right knees of the rats were surgically destabilized by medial collateral ligament and meniscus transection while the left knees remained untreated as a control group [8, 9]. Before and for two days after surgery the animals received Rimadyl *s.c.* (5 mg/kg body weight, Pfizer Inc., USA) as analgetic. The rats were subsequently anesthetized using a combination of ketamine (80 mg/kg body weight, WDT, Germany), acepromazine (1 mg/kg body weight, Ceva Sante Animale, France) and xylazine (5 mg/kg body weight, Bayer AG, Germany) *i.p.*. Afterwards, selecting a medial approach the transection of the medial *ligamentum collaterale* and partial resection of the medial meniscus was performed (Fig. 1 a1-a3). For the following 8 weeks the animals underwent a forced mobilization procedure on a self-constructed rotor disc apparatus (Fig. 1b). After 6 weeks the animals, randomly divided into two groups by picking numbers out of a hat, were either treated with 100 μL dPGS (30 mg/kg body weight) or with saline (0.9 %, Fresenius Kabi GmbH, Germany). Both was administered subcutaneously once a day for 2 weeks. After 8 weeks the animals were sacrificed. Furthermore, the animals were weighed each week over the course of the whole experiment.

### Footprinting analysis

At specific points in time (week 1, 5 and 7) a footprinting analysis was done to measure the influence of OA and the treatment on stride length and step width of the animals. The rat’s hind paws were inked and the animals were encouraged to walk over a white paper walking track.

### Decalcification and paraffin embedding of rat knee joints and organs

The explanted joints were fixed using 4 % PFA solution for three days and decalcified in formic acid (5 %, Carl Roth, Germany). Then they were incubated with ammonia solution (Merck KGaA, Germany) for 30 minutes to ensure neutralization and afterwards rinsed in water for 24 h. After rinsing in water, the samples were dehydrated using an ascending ethanol series and embedded in paraffin. To assess a possible accumulation of dPGS in organs responsible for drug elimination liver, spleen and kidneys were explanted.

### Histological staining and scoring

Deparaffinized sections (3–5 μm) were Haematoxylin Eosin (HE) stained using a standard protocol. For Alcian Blue (AB) staining, the sections were incubated for 3 minutes in 1 % acetic acid and then stained 15 minutes in 1 % AB (Carl Roth GmbH). Subsequently, they were rinsed in 3 % acetic acid and cell nuclei counterstained using nuclear fast red aluminium sulfate solution (Carl Roth GmbH) for 5 minutes. For the safranin-O staining the slides were incubated for 10 minutes in a Weigert’s iron hematoxylin (Carl Roth GmbH) and afterwards rinsed in running tap water for 10 minutes. Subsequently, the sections were stained in 0.001 % fast green solution (Sigma-Aldrich) for 5 minutes. After rinsing them briefly in 1 % acetic acid the slides were stained in 0.1 % safranin-O solution (Merck KGaA) for up to 5 minutes. Dehydrated sections were mounted with Entellan mounting medium (Merck KGaA). The stained sections were further analyzed by applying score systems by two observers. To consider species-dependent peculiarities in rats two scoring systems for OA were used, the well-established Mankin Score, which was originally designed for human cartilage and the score system adapted by Glasson et al., [10] suitable for the very thin rodent joint cartilage. The scoring system according to Krenn et al., [11] was applied to classify synovitis.

### Statistical analysis

Data were expressed as mean values with standard deviation and analyzed using one sample *t* test, one-way ANOVA with a *post-hoc* Tukey Test (GraphPad Prism 5, GraphPad software Inc, USA) and the Mann Whitney U Test. The GRUBB’s test was performed to identify outliers. If applicable, the Kolmogorov-Smirnov test was used to prove the presence of a Gaussian distribution. Statistical significance was set at a *p* value of ≤ 0.05.

## Results

### Characterization of primary adult rat articular chondrocytes

During further culturing the rat articular chondrocytes gained a more flattened and fibroblast-like appearance. Nevertheless, they still expressed the typical cartilage marker type II collagen and the master chondrogenic transcription factor sox9 (Additional file 1: Figure S1). Type I collagen which is an indicator of dedifferentiation could also be detected (Additional file 1: Figure S1). The immunolabelling for both collagen types was detected extracellularly localized at extracellular matrix fibrils and intracellularly in the perinuclear rER region. The expression of type II collagen and the nuclear signal for sox9 did not decrease during the first passage. Compared with the start of culture (passage 0) the visible F-actin fiber bundles increased. A colocalization of vinculin immunolabeling at the ends of F-actin fiber bundles indicated focal adhesion sites (Additional file 1: Figure S1).

### Uptake and localization of dPGS in rat articular chondrocytes

**Fig. 2:**
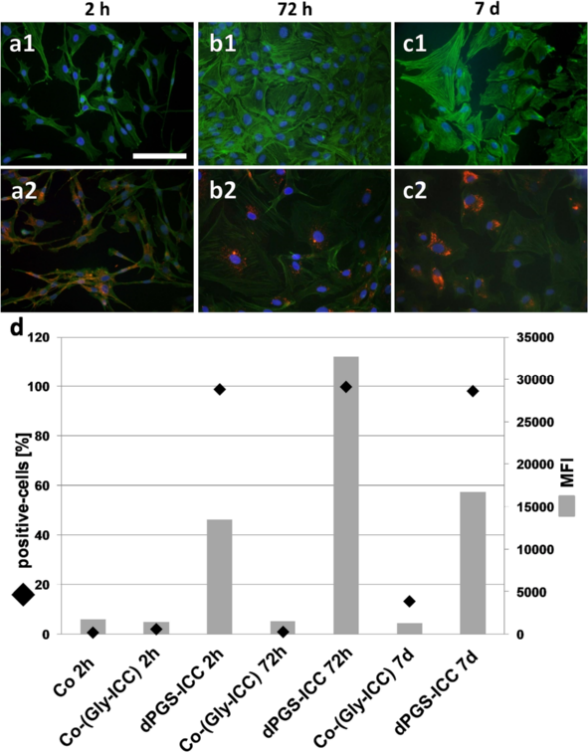
**a1-d** Accumulation of nanoparticular compounds (dPGS) in cultured rat chondrocytes. Glass cover slides were seeded with rat articular chondrocytes and incubated with respective glycerol-ICC (10^−6^ mol/L) as a control group (**a1-c1**) or dPGS-ICC (10^−6^ mol/L) (**a2-c2**). Representative images were taken 2 hours (**a1, a2**), 72 hours (**b1, b2**), and 7 days (**c1, c2**) after stimulation. Depicted are phalloidin-Alexa488 (green) as cytoskeleton staining, DAPI (blue) as the nucleus staining and ICC coupled dPGS (red) to visualize the accumulation and localization of dPGS (seeding density: 15.600 cells/cm^2^, cell passage: 4). Scale bar: 100 μm. **d**: Rat articular chondrocytes of three animals were pooled and incubated for 24 hours with dPGS-ICC or respective glycerol-ICC as a control group (both: 10^−6^ mol/L). One control was left untreated to be used as an isotype control. The cells were evaluated flow cytometrically. Depicted are the mean number of cells tested positive for dPGS uptake as percentage (data points) and the mean fluorescence intensities of dPGS-ICC inside the cells (MFI [mean fluorescence intensity], bars) (events counted: 10.000, seeding density: 20.000 cells/cm^2^, cell derived from 4th passage). A representative experiment is shown

The incubation of isolated articular chondrocytes of rats with dPGS-ICC (c = 10^−6^ mol/L) led to a strong fluorescent signal after 2 hours (Fig. 2a2) which increased over the course of the next 72 hours (Fig. 2b2) and even after 7 days (Fig. 2c2). Furthermore, a perinuclear aggregation of dPGS-ICC was detected after 7 days. Controls, incubated with glycerol-ICC did not exhibit any ICC-signal (Fig. 2a1, b1 and c1). The cytoskeletal actin was more intense stained in the untreated controls compared with the dPGS treated samples (Fig. 2).

### FACS based uptake and release study of dPGS-ICC by rat-derived articular chondrocytes

All dPGS-ICC treated chondrocytes showed dPGS associated fluorescence (Fig. 2d). After 2 hours nearly 100 % of chondrocytes were positive for dPGS uptake which could also be observed after 72 hours and 7 days. Nevertheless, the mean fluorescence intensity (MFI) after 72 hours nearly doubled in comparison with the amount found after 2 hours or even 7 days. After 7 days the MFI decreased to nearly the amount of the MFI after 2 hours. Nevertheless, nearly 100 % of cells were still positive for dPGS.

### Histological staining of organs

**Fig. 3:**
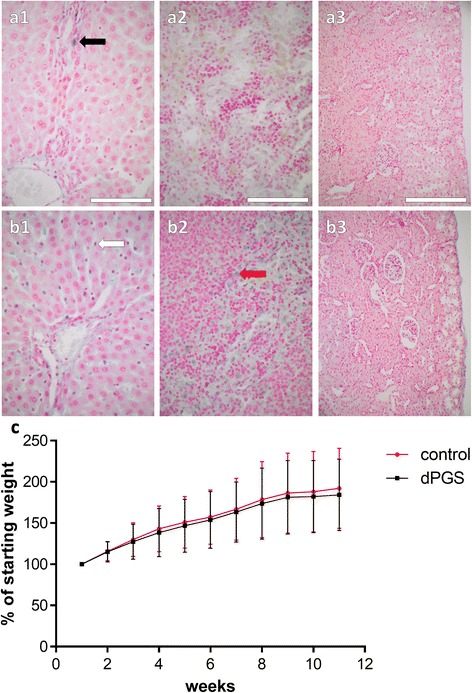
**a**-**c** Comparison of untreated rats and rats treated with dPGS concerning detectable dPGS deposition in the liver, spleen and kidney and development of body weight in adult male Wistar rats over the course of 11 weeks. **a**-**b**: Paraffin sections of organs derived from male adult Wistar rats were stained using AB. Depicted are the remaining dPGS nanoparticular compounds in the liver, spleen and kidney of the treated rats (**b1**-**3**) in contrast to untreated rats (**a1**-**3**). Scale bars: 200 μm (**a1**-**2**, **b1**-**2**) and 100 μm (**a3**, **b3**). (**a1**) black arrow: mast cell, (**a2**) white arrow: Kupffer cell, (**b2**) red arrow: spleen macrophage. **c**: Depicted are the control animals and the animals treated with dPGS (*n* = 12). The weight is depicted as the percentage of weight in comparison to the weight at the beginning

Organs of the treated and untreated rats revealed no macroscopical degenerative alterations. To assess a possible accumulation of nanoparticular dPGS and subsequent toxic changes in organs liver, spleen and kidneys of treated and untreated rats were stained with HE and AB. In dPGS treated rats the HE staining revealed no histopathological alterations of liver, spleen and kidney (Fig. 3a-b). In the dPGS treated group but also in the control animal group few cells in the connective tissue areas of the liver which surround the vessels (the so-called Glisson trias) could be positively stained by AB suggesting the presence of heparin containing mast cells. Additionally, but only in the dPGS treated group some other cells were AB positive (Fig. 3a1, b1). These dPGS containing cells were mostly localized nearby the so-called space of Disse, probably representing the phagocyting Kupffer cells. However, the hepatocytes were generally AB negative. Some staining which was not cell-associated could also be detected in the extracellular matrix of the faint connective tissue layer surrounding the central veins (not shown). The spleen revealed also several AB positive cells which could only be detected in the dPGS treated animal group (Fig. 3a2, b2). These cells, probably spleen macrophages were localized in the red pulp whereas the white pulp of the spleen was AB negative. The cortex of the kidney was completely devoid of AB positivity (Fig. 3a3, b3). The inner medulla revealed a slight staining in both groups due to the extracellular content of sulfated glycosaminoglycans there (not shown).

### Changes in body weight, stride length and step width between studies groups

**Fig. 4:**
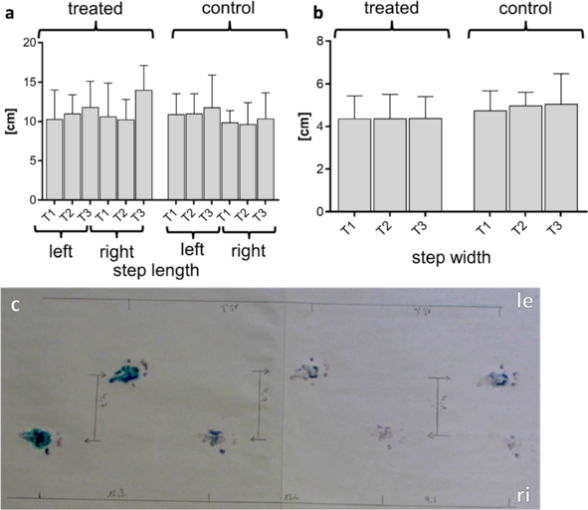
**a**-**c** Comparison of stride length, step wide between treated and untreated rats and footprinting analysis. At defined points in time the rats’ hind feet were inked and they were encouraged to walk over a white strip of paper. The stride length (**a**) and step width (**b**) of 12 adult male Wistar rats (*n* = 6: treated; *n* = 6: untreated) was measured before surgical procedure (T1), 6 weeks after surgical treatment, before start of treatment (T2) and before finalization, after two weeks of treatment (T3). A representative exemplary image of the measurement procedure is shown. The image shows the gait pattern of an animal prior to surgery (**c**). le: left, ri: right leg

The rats exposed no clinical signs of OA such as reduced activity, joint pain, −swelling or any visible gait alterations. The body weight of animals, dPGS treated or not, did not significantly change (Fig. 3c). The footprinting analysis revealed that, although the animals had severe osteoarthritic changes in medial compartments of their knees none of it led to a significant change in stride length or step width (Fig. 4a-c).

### Histological staining of joint cartilage

**Fig. 5:**
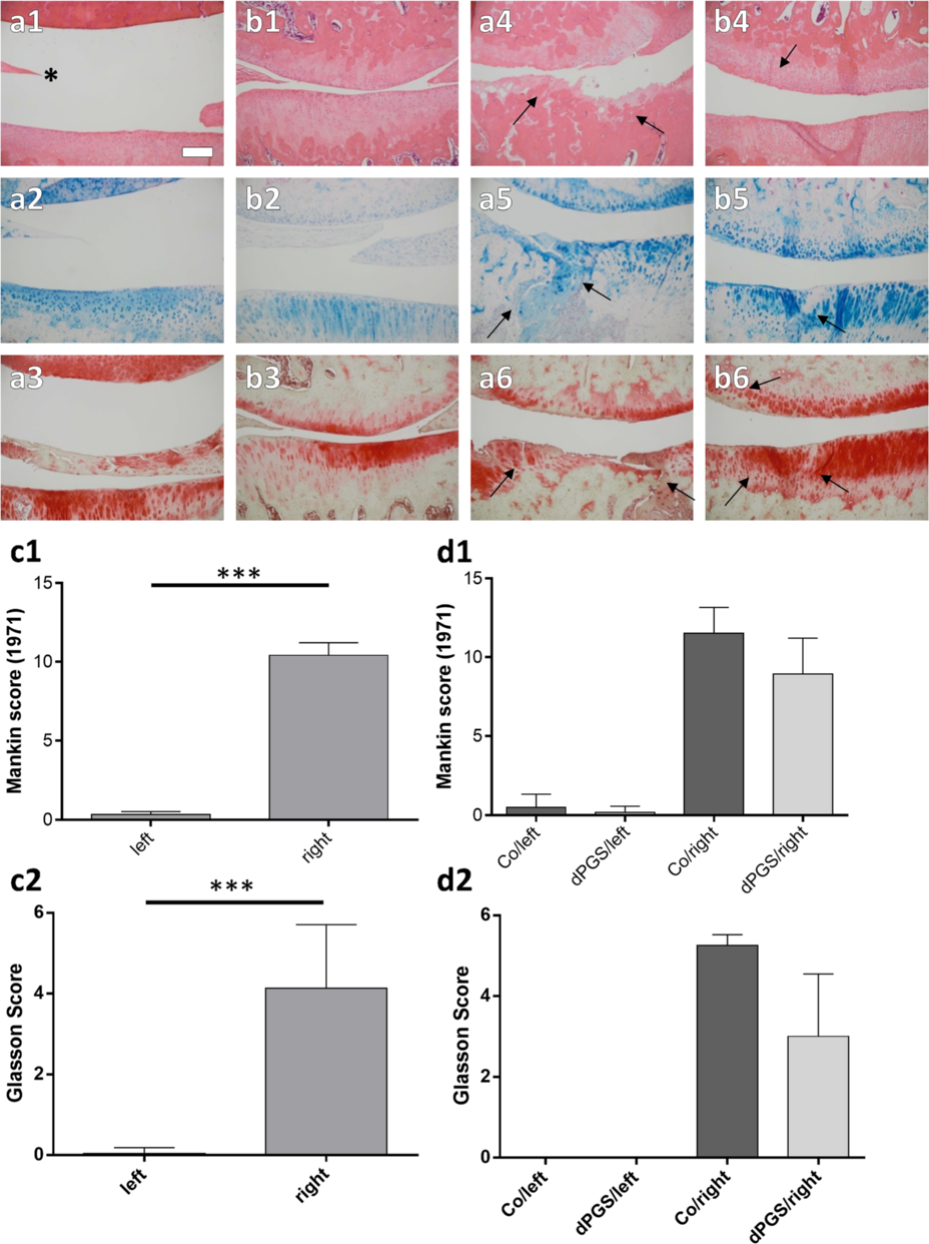
**a**-**d** Joint histology: comparison of untreated rats (**a**) and rats treated with dPGS (**b**) as well of histological scoring results (**c**, **d**). Representative frontal cross sections of adult male Wistar rat joints are shown. Depicted is the joint space between femur condyle (top) and the tibia plateau (bottom). Shown are the not surgical destabilized left knees of untreated (**a1**-**3**) and treated rats (**b1**-**3**). Furthermore, the surgical destabilized right knee of untreated (**a4**-**6**) and those of dPGS treated animals (**b4**-**6**) are depicted. The tissue was stained using HE (**a1**, **b1**, **a4**, **b4**), AB (**a2**, **b2**, **a5**, **b5**) or safranin-O staining (**a3**, **b3**, **a6**, **b6**). * = meniscus. The regions of interest are labeled by black arrows. Scale bar 200 μm. HE and safranin-O stained paraffin sections of rat knee joints were evaluated using the score systems according to Mankin (1971) (**c1**, **d1**) and Glasson et al., (2010) (**c2**, **d2**). Depicted are the differences of the left (not surgically altered) and the right knees (surgically destabilized) of all animals (*n* = 12) (**c1**-**2**). Furthermore, the differences between left knees and the right knees for the untreated control and the treated animals are shown separately (**d1**-**2**). *n* = 6 per group. ***p < 0.001

At the macroscopical level no changes of the joint surface or osteophyte formation could be detected. The rat knee joints of the left legs which were not surgically destabilized showed almost no signs of OA, neither in the untreated (Fig. 5a1-a3) nor in dPGS treated rats (Fig. 5b1-b3). The surfaces of the cartilage layer was almost completely smooth; no major fibrillation or other degenerative features were detectable at all, an equal chondrocyte distribution and an intact tidemark was detected (Fig. 5a1-b3). Furthermore, the stainings of AB and safranin-O were distributed within the zones as expected for healthy rat articular cartilage. The joint cartilage of the right knees of the rats showed significantly different results (Fig. 5a4-a6, b4-b6) than their left counterparts. The tidemark was not any longer detectable and the cartilage was severely disintegrated in the medial joint compartiment (Fig. 5a4). Large areas of cartilage completely devoid of cells could be found indicating hypocellularity (Fig. 5a4-a6) and in some areas of the chondrocytes formed clusters and even subchondral cysts were found. In several cases cartilage fissures extended deeply into the calcified zone of the joint cartilage. Furthermore, the safranin-O staining intensity was severily reduced or a focally complete disintegration of cartilage extracellular matrix was discernible (Fig. 5a6). In contrast to the untreated animals, rats treated with dPGS revealed an overall less damaged cartilage (Fig. 5b4-b6). Damage of the cartilage structure remained for most of the samples restricted to the cartilage surface. Only few cases with deeper joint cartilage fissures were found. Accordingly, the proteoglycan staining intensity and distribution in the joint cartilage of the rats treated with the dPGS remained mostly as usually expected under physiological conditions.

### Osteoarthritis score analysis of treated and untreated rats

**Fig. 6:**
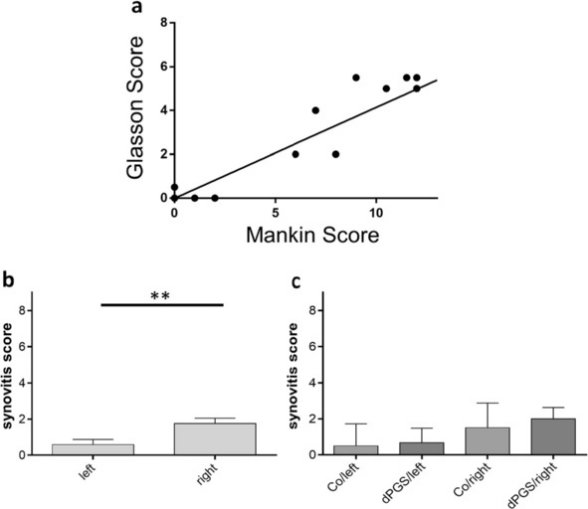
**a**-**c** Correlation between both OA scoring systems and assessment of the synovitis using the score according to Krenn et al., (2006). Correlation between both scoring systems (**a**). Depicted are the differences of the left (not surgically altered) and the right knee (surgically destabilized) of all animals (*n* = 12) (**b**). Furthermore, the differences between left and the right knees for the untreated control and the treated animals are shown separately (**c**). *n* = 6 per group. **p < 0.01

Evaluation of HE and safranin-O stained tissue sections of treated and untreated rats was undertaken by applying the Mankin score [12] and the scoring system proposed by Glasson et al., [10] to them to monitor osteoarthritic alterations. The left (not surgically destabilized) and the right (surgically destabilized) knee joints of the rats were highly significant different (*p* < 0.0001) when comparing all animals of both groups (treated with dPGS or not). The right knees showed a significantly higher score proving the onset of OA than in the left knees underlined by the results of both scoring systems (Fig. 5c1-2). The difference between the articular cartilage of the left knees of rats treated with dPGS compared with those only received saline injections was not significant. The comparison of the right knees of dPGS-treated and untreated animals revealed that the Mankin and Glasson score of treated animals was lower than the score of placebo treated rats, suggesting their OA is less severe than the ones of untreated rats (Fig. 5d1-2). Mankin and Glasson scoring results of all animals revealed a significant correlation (Fig. 6a). However, the difference did not reach the significance level. Additionally, we scored the synovial membrane of the joint capsule using the score developed by Krenn et al. [11]. We found a significant score difference between the left (control) and right (OA) joint (Fig. 6b), while there was no difference visible between the untreated and treated groups of left joints (Fig. 6b) and neither between the right joints (Fig. 6c).

## Discussion

Due to the thin cartilage layer in rats the isolation of adult rat articular chondrocytes derived from the knee joint is challenging and only low cell numbers can be harvested. Since it is well known that human and also rat derived chondrocytes lose their specific differentiated phenotype during cell expansion and further culturing in monolayer a process called dedifferentiation [13–15], the expression of cartilage markers was evaluated in the present study. Cartilage-specific markers such as type II collagen and sox9 could be demonstrated indicating that the primary rat chondrocytes introduced in the experiments exhibited still differentiated functions. Previous studies indicated that chondrocytes can maintain their differentiated phenotype for up to four passages in monolayer culture [13, 14]. Cultured rat chondrocytes easily took dPGS up as an important precondition for using dPGS in the joint. In another study were viable human cartilage chips were tested for penetration by dPGS we found that dPGS could also penetrate the cartilage extracellular matrix to some degree (50 μm distance). The results of the FACS analysis suggest that dPGS is not only taken up by the cells over the course of the experiment but might also be actively segregated again back in the culture supernatant. However, this assumption requires further experimental support. An instability OA model of the rat knee was selected in the present study to test the effect of dPGS on OA progression. This OA model has already been used by various research groups before [16–18]. Since OA needs several weeks for manifestation in this model, the daily dPGS treatment was started after 6 weeks and lasted for two weeks. The treatment had no effect on overall behavior, body weight and gait of the rats indicating no clinical signs of OA. To exclude toxic effects of the nanoparticular compounds during this rather long treatment course we analyzed the histology of organs involved in metabolism and elimination of toxic compounds such as liver, spleen and kidney. The AB staining was used here to detect negatively charged dPGS particles [19] and therefore, to monitor a potential accumulation of dPGS within these organs. It is known that administered nanoparticular compounds can accumulate in secondary target organs [20, 21]. Some cross reaction with heparin can usually be found using this staining procedure due to its negative charges and similar chemical composition like dPGS. Therefore, mast cells which contain cytoplasmic heparin inclusions were also stained positively blue with AB and could be detected in the connective tissue of the liver surrounding the Glisson trias vessels in all animals. In addition to these mast cells, the dPGS treated animals but not the controls revealed also dPGS positive Kupffer cells. This observation is supported by the study of Holzhausen, Gröger et al., [19]. Apart from this observation, the dPGS treatment did not induce any histopathological changes in the investigated organs suggesting that it might not impair organ function at the administered dose and time course. In agreement, some macrophages in the spleen revealed dPGS in the dPGS treated rats, but not in the controls. However, dPGS could not be shown in the kidneys. This observation suggests that the parenchymal cells are able to eliminate the dPGS in response to a daily treatment and a longer treatment with dPGS could be possible. The onset of OA could be proven in all animals included in this study, since there was a highly significant difference in the Mankin Score and also in the Glasson score values between the operated right and unoperated left knee joints. The difference between the right knee joints of the group treated with dPGS *versus* the controls was clearly detectable and supported by histopathology but it did not reach the significance level. Therefore, it suggests that the dPGS treatment led to an improvement in the condition of the knee joints of the treated animals. Furthermore, the fact that the animals treated with dPGS also showed a lower Mankin score value than the untreated animals in their left knees, which were not surgically destabilized underlines chondroprotective capabilities of dPGS. The grade of synovitis was significantly higher in operated compared to the unoperated legs of the animals suggesting that OA progression was generally associated with synovitis in this rat model. Nevertheless, the differences between dPGS treated animals and controls were not significant. However, neither clinical signs of OA such as pain, joint swelling, warmness and fluctuation nor macroscopical alterations could be found during joint explantation. The mode of action of dPGS on joint inflammation remains a matter of debate. Studies in other inflammatory models revealed a suppressive effect of dPGS on NF-κB and AP-1 activation. These both transcription factors are involved in pathways leading to the release of inflammatory cytokines [22, 23] and other mediators or enzymes such as matrix-metalloproteinases [24]. Apart from its modulatory effect on IL-10 and TNFα [16], dPGS affects also complement factors e.g. C5a and selectins thereby impairing leukodiapedesis [5]. Several tissues interact with each other under OA conditions [25]: apart from articular cartilage, synovial membrane, infrapatellar joint fat pad and subchondral bone cross-talk [26, 27]. Therefore, the mode of chondroprotective action of dPGS requires further investigations.

This study has some limitations. Since we did not detect clinical features of OA one should consider a more sensitive testing system for gait analysis such as the so called “cat walk” which is an automated quantitative gait analysis system for rodents in the future. Furthermore, a sensitive pain tests could be included. Since dPGS treatment led generally to less severe histopathological OA in the rats, more effective treatment profiles should be selected (earlier start or longer duration of dPGS treatment) in the future.

## Conclusions

The present study indicates a high cytocompatibility and a rapid uptake of dPGS in cultured rat chondrocytes. The Mankin and Glasson histological score values were lower in OA rats treated with dPGS compared with control animals. The analyzed organs revealed no degenerative changes due to dPGS accumulation suggesting biocompatibility. Taken together, the results indicate a chondroprotective and anti-inflammatory potential of dPGS in the osteoarthritic knee joint suitable to suppress OA progression.

## Additional file

Additional file 1: Figure S1a1-e2. Characterization of cultured rat primary knee joint chondrocytes. Primary chondrocytes immediately after isolation (passage 0, upper row, a1-e1) and in the first passage (lower row, a2-e2) are depicted light microscopically (a1-2) and were characterized for the expression of type II collagen (green, b1-2), type I collagen (green, c1-2), sox9 (green, d1-2) and vinculin (red)/F-actin (green) (e1-2). Cell nuclei are counterstained using DAPI (blue). a1-2: scale bar 100 μm, b1-e2: scale bar 50 μm. (TIF 7376 kb)

## Abbreviations

AB: alcian blue
DAPI: 4’, 6-diamidino-2-phenylindole dihydrochloride
dPGS: dendritic polyglycerol sulphate
FACS: fluorescence activated cell sorting
FCS: fetal bovine serum
HE: Haematoxylin Eosin
IL-1: interleukin-1
MFI: mean fluorescence intensity
OA: osteoarthritis
PBS: phosphate buffered saline
PFA: paraformaldehyde
s.c.: subcutaneous
TBS: Tris-buffered Saline
TNFα: tumor necrosis factor
