# Supplementary figures and images for: Influence of dendritic polyglycerol sulfates on knee osteoarthritis: an experimental study in the rat osteoarthritis model
Source: BMC Musculoskelet Disord. 2015 Dec 15;16:387. doi: 10.1186/s12891-015-0844-3 (PMC4681118; doi:10.1186/s12891-015-0844-3)

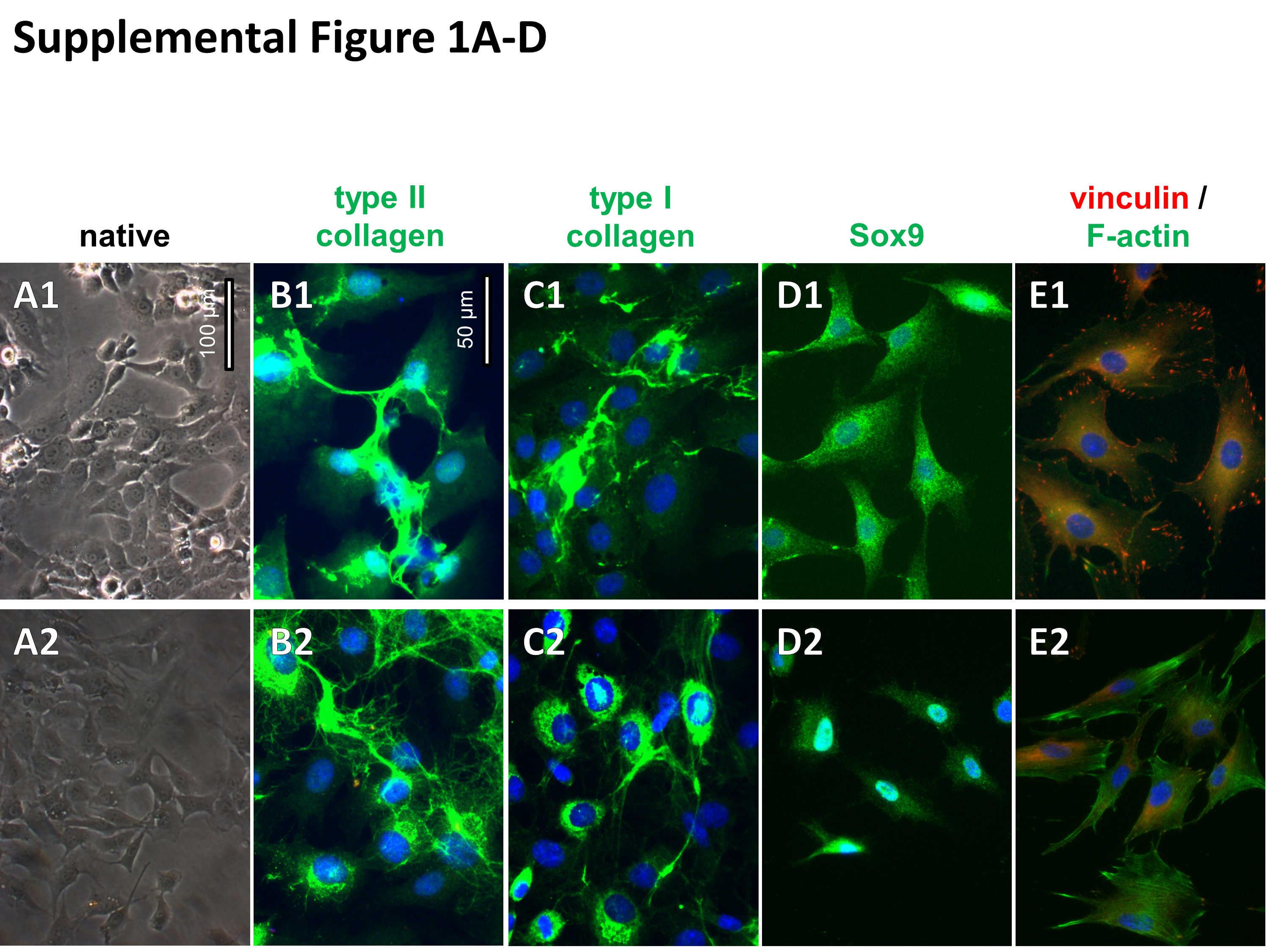

Supplement: Additional file 1: Figure S1a1-e2. — Characterization of cultured rat primary knee joint chondrocytes. Primary chondrocytes immediately after isolation (passage 0, upper row, a1-e1) and in the first passage (lower row, a2-e2) are depicted light microscopically (a1-2) and were characterized for the expression of type II collagen (green, b1-2), type I collagen (green, c1-2), sox9 (green, d1-2) and vinculin (red)/F-actin (green) (e1-2). Cell nuclei are counterstained using DAPI (blue). a1-2: scale bar 100 μm, b1-e2: scale bar 50 μm. (TIF 7376 kb) [file 12891_2015_844_MOESM1_ESM.tif]
